# Soluble P2X7 receptor (sP2X7R) as a marker of inflammation in pregnancy

**DOI:** 10.1007/s11302-026-10186-y

**Published:** 2026-09-11

**Authors:** Valentina Vultaggio-Poma, Selene Schio, Cristina Taliento, Rosa Galasso, Simonetta Falzoni, Claudia D’Angiolillo, Sara Ghisellini, Pantaleo Greco, Francesco Di Virgilio, Anna Lisa Giuliani

**Affiliations:** 1 Department of Medical Sciences, University of Ferrara, Ferrara, Italy; 2 Unit of Obstetrics and Gynaecology, Sant’Anna University Hospital, Ferrara, Italy; 3 Unit of Clinical Pathology, Sant’Anna University Hospital, Ferrara, Italy

**Keywords:** Pregnancy, sP2X7R, Inflammation, Maternal-fetal interface, Biomarker, Pregnancy-related disorders

## Abstract

The purinergic P2X7 receptor (P2X7R) is a key regulator of inflammation and pro-inflammatory cytokine release. Recently, a soluble form of P2X7R (sP2X7R) has been identified in the circulation of healthy individuals and significantly increased in patients with inflammatory disorders. As pregnancy is characterized by finely regulated inflammatory adaptations and considered a state of low-grade inflammation, we hypothesized that P2X7R may contribute to the physiological inflammatory status of pregnancy and to pregnancy-related inflammatory disorders. A cohort of 90 pregnant women was evaluated at the first, second, and third trimester of pregnancy, and 30 age-matched non-pregnant women were used as controls. We measured sP2X7R blood concentrations, and the neutrophil-to-lymphocyte ratio (NLR) was used as a reference inflammatory marker. Associations with maternal characteristics, pregnancy complications and foetal sex were investigated. Serum sP2X7R concentrations were significantly higher in pregnant than in control women and increased progressively throughout pregnancy. Although sP2X7R and NLR showed similar trends, sP2X7R provided complementary information. First-trimester sP2X7R concentrations were significantly higher in multigravida women, in those with gestational hypothyroidism, and in those carrying a male foetus. No significant differences were observed according to Body Mass Index (BMI), gestational diabetes, or gestational hypertension. Furthermore, sP2X7R was detectable in umbilical cord blood and positively correlated with maternal circulating concentrations. To our knowledge, this is the first study to show that circulating sP2X7R increases during normal pregnancy and reflects the physiological inflammatory adaptations of gestation. The results indicate that circulating sP2X7R is a potential biomarker of inflammatory responses and maternal-fetal immune interactions during pregnancy.

## Introduction

Inflammatory processes are involved in every stage of fertility, from the menstrual cycle to pregnancy and later during labour [1, 2]. During pregnancy, the establishment and maintenance of immune tolerance at the maternal-fetal interface are critical for a successful outcome. The maternal immune system undergoes substantial changes, allowing acceptance of the semi-allogeneic fetus while preserving the ability to combat invading pathogens [3, 4]. Compared to the non-pregnant state, normal pregnancy is characterized by a mild increase in serum levels of both pro- and anti-inflammatory cytokines [5–7].

It has been proposed that a normal pregnancy consists of three distinct immunological stages corresponding to the three trimesters. The initial pro-inflammatory stage, marked by a Th (T helper)1-driven response with high levels of cytokines such as Interferon (IFN)-γ, Interleukin (IL)−2, IL-12, and Tumor Necrosis Factor (TNF)-α, supports implantation and placentation. The subsequent anti-inflammatory period of fetal development is characterised by a shift towards a Th2-dominant environment with elevated levels of IL-4, IL-5, IL-10, and IL-13. In the third trimester, the immune system returns to a pro-inflammatory state that supports labour and parturition, indicated by increased levels of IL-6, IL-8, and IL-10 [8].

Deregulation of this “physiological” inflammatory response may trigger adverse effects on pregnancy outcome [9–12]. Several studies show that excessive elevations of pro-inflammatory cytokines in maternal serum and amniotic fluid are implicated in the risk of preterm delivery [11, 13], miscarriage [14], gestational hypertension [15, 16], gestational diabetes [17] and preeclampsia [18–20].

The purinergic P2X7 receptor (P2X7R), an ATP-gated ionotropic receptor with a central role in inflammation and immunity [21, 22], has been implicated in several diseases, ranging from infections to neurodegeneration, and from autoimmune diseases to cancer [23]. The P2X7R, which is an ubiquitary receptor, highly expressed on myeloid immune cells, primarily activates the NLRP3 inflammasome, provoking the release of pro-inflammatory cytokines [21]. Furthermore, P2X7R expression in uterine epithelial cells and placenta has been reported to be involved in different pregnancy outcomes. Several studies have shown that activation of the P2X7R/NLRP3 pathway is involved in the pathogenesis of preeclampsia, preterm birth and other pregnancy syndromes associated with placental inflammation [24, 25].

Recently, a shed form of the P2X7R (sP2X7R) has been detected in peripheral blood, partly associated with circulating microparticles (MPs), which showed a correlation with C-reactive protein (CRP) levels [26]. sP2X7R blood concentrations were found to be increased in various inflammatory conditions such as temporal lobe epilepsy [27], Alzheimer’s disease [28], and obesity [29], and correlated with COVID-19 progression and outcomes [30, 31].

Based on these observations, and considering the inflammatory status associated with pregnancy, we hypothesized that sP2X7R is involved in intercellular communication at the maternal-fetal interface and that measuring its levels throughout gestation might help distinguish healthy pregnancies from those that develop inflammatory complications. In this study, we therefore measured sP2X7R serum concentrations in a cohort of pregnant women across all three trimesters in various pregnancy conditions. As a reference inflammatory marker, we evaluated the neutrophil-to-lymphocyte ratio (NLR) [32, 33] since other inflammatory parameters, such as C reactive protein (CRP), are not included in the panel of tests routinely performed during pregnancy.

## Materials and methods

### Participants and study design

We initially recruited 100 pregnant women at the Unit of Obstetrics and Gynaecology, Sant’Anna University Hospital (Ferrara, Italy), starting in May 2023. Participants were enrolled during obstetric visits, and blood samples were collected during antenatal care in the first (0–12 weeks’ gestation), second (13–27 weeks’ gestation), and third (> 28 weeks’ gestation) trimesters, as well as at delivery. Blood samples were also collected from umbilical cords at birth. Gestational age was calculated from the last menstrual period.

Inclusion criteria were maternal age of at least 18 years, singleton pregnancy, and first-trimester gestation. Exclusion criteria included known fetal congenital anomalies at the time of consent and participant conditions that made enrolment difficult, such as drug addiction or psychiatric disorders. Control serum samples were obtained from 30 healthy, non-pregnant, volunteer age-matched women. Written informed consent was obtained from all participants before sample collection. The protocol was approved by the Ethical Committee of the Ferrara district (Study CE-AVEC 591–2023-Oss-AOUFe).

Blood samples were collected in vacutainers with or without K2EDTA (BD Vacutainer), and whole blood samples were used to measure routine haematological parameters at the Clinical Pathology Laboratory of Sant’Anna University Hospital using standard methods. Serum samples were obtained by centrifugation at 4 °C for 15 min at 1000 × g, aliquoted, and stored at − 80 °C until use. Freezing and thawing cycles were carefully avoided.

### Measurement of serum sP2X7R

sP2X7R concentration was measured in serum samples using the Human P2X purinoceptor 7 (P2RX7) ELISA kit (CSB-EL017325HU Cusabio, Houston, Texas, USA) following the manufacturers’ instructions. Optical density was measured at 450 nm with wavelength correction at 570 nm with a Multiskan FC spectrophotometer (Thermo Scientific, Waltham, MA, USA).

### Statistical analysis

Statistical analyses were performed using GraphPad Prism 10.0.1 software (San Diego, CA, USA). Data are shown as means ± SE. Comparisons between two independent groups were performed using the nonparametric Mann–Whitney U test. Because of some missing samples, comparisons among multiple groups were performed using one-way ANOVA mixed-effects analysis followed by Tukey’s multiple test to compare sP2X7R levels between each time. Spearman's rank correlation was used to assess the bivariate association between the parameters of interest. A *p*-value ≤ 0.05 was considered statistically significant.

## Results

### Maternal characteristics

One hundred pregnant women, aged 20 to 45 years (mean 33 ± 4.8 years), were recruited between May and July 2023 at the Obstetrics and Gynaecology Unit of Sant’Anna University Hospital. Maternal demographic and clinical data are presented in Table 1. The women were followed throughout all three trimesters of pregnancy and at delivery, and their blood samples were collected to measure serum sP2X7R concentrations. Among the most common pregnancy complications, gestational diabetes occurred in 12 women and hypertension in 8; 16 developed gestational hypothyroidism, 15 were overweight, and 14 were obese. None of the pregnant women developed pre-eclampsia. Ten participants were excluded from the analyses due to spontaneous abortion (*n* = 2), voluntary termination of pregnancy (*n* = 1), intrauterine fetal death (*n* = 2), or transfer of care to another birth centre (*n* = 5). The final study population therefore consisted of 90 patients. In addition, some biological samples from these 90 patients were not available for all trimesters of pregnancy. We also measured sP2X7R concentrations in the serum of 30 healthy, non-pregnant women (control group, mean age 32 ± 4.5 years) whose serum CRP levels were below 0.5 mg/dL and were therefore considered free from any inflammatory state.

**Table 1:** Maternal demographic and clinical data

Demographic and clinical data	Mean (SD)
Age (Years)	33 (4.8)
Pre-pregnancy BMI (kg/m^2^)	24.6 (4.7)
Systolic blood pressure after 20 weeks (mmHg)	118 (12.3)
Diastolic blood pressure after 20 weeks (mmHg)	71 (8.2)
Trimester I sample (weeks)	10 (1.7)
Trimester II sample (weeks)	22 (2.8)
Trimester III sample (weeks)	31 (3.0)
Gestational age at delivery (weeks)	39 (1.3)
Birthweight (grams)	3196 (0.7)
	N (%)
Primigravida	37 out of 90 (41.1)
Multigravida	53 out of 90 (58.9)
Nulliparous	51 out of 90 (56.6)
Multiparous	38 out of 90 (42.2)
Previous miscarriage	23 out of 90 (25.5)
Gestational diabetes	12 out of 90 (13.3)
Gestational Hypertension	8 out of 90 (8.8)
Hypothyroidisms	16 out of 90 (17.7)
Overweight (BMI 25—30)	15 out of 88 (17.0)
Obese (BMI > 30)	14 out of 88 (15.9)
Vaginal delivery	65 out of 88 (73.9)
Cesarean delivery	23 out of 88 (26.1)
Fetal sex, female	37 out of 83 (44.6)
Fetal sex, male	46 out of 83 (55.4)

### sP2X7R concentrations increase during pregnancy

**Fig. 1:**
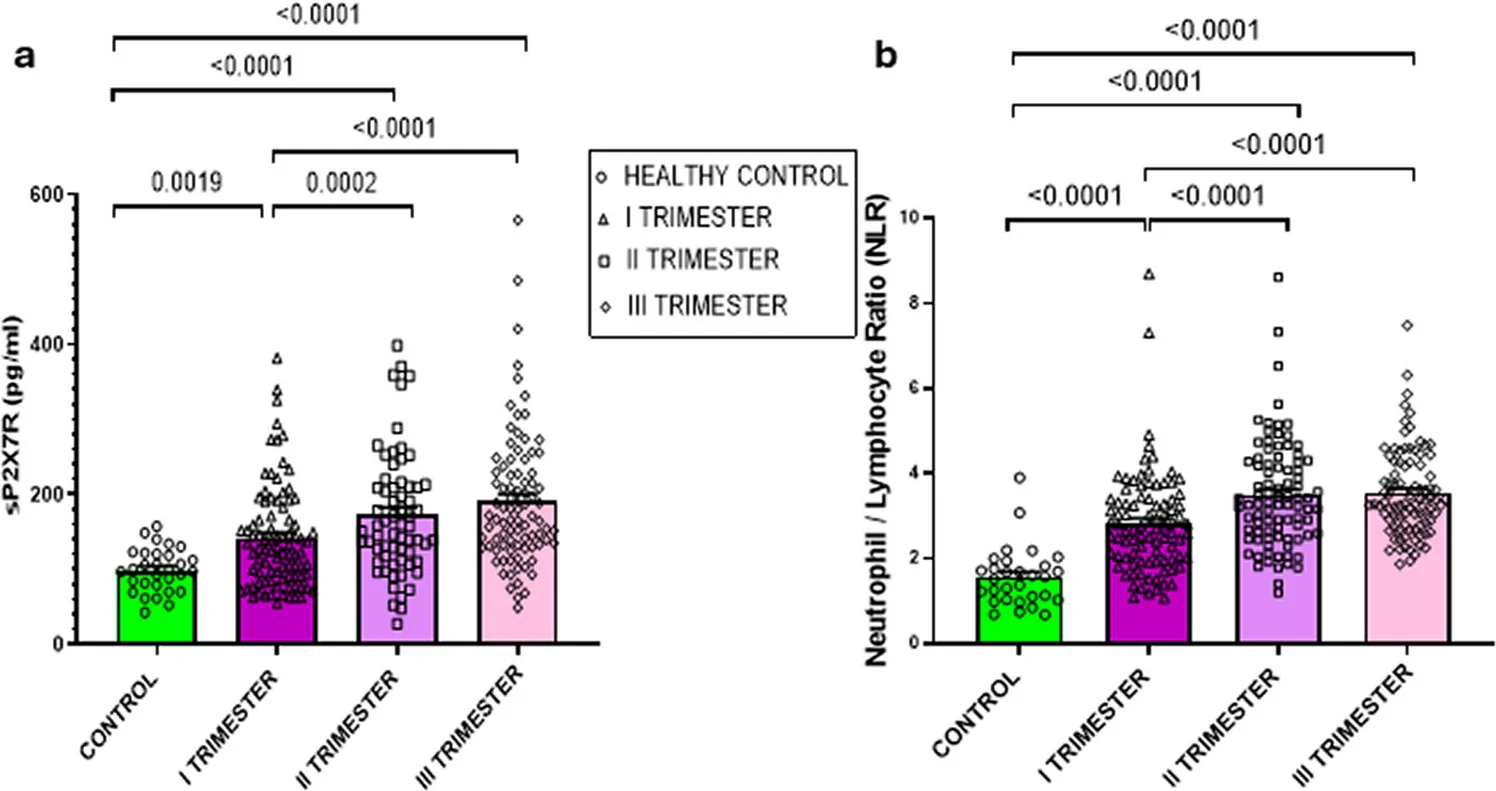
Serum sP2X7R concentrations and neutrophil-to-lymphocyte ratios (NLR) during pregnancy. Pregnant women were submitted to blood withdrawal at each trimester and serum sP2X7R and NLR were determined as described under methods. **a** Serum sP2X7R concentrations and **b** NLR values in women at the I, II, and III trimester of pregnancy and in non-pregnant healthy control women. sP2X7R ELISA measurements were performed in technical duplicates. Data are expressed as mean ± SEM. Statistical analysis was performed as described under Methods. Differences were considered statistically significant when *p* < 0.05

The mean amount of serum sP2X7R in the non-pregnant, healthy women was 98.9 ± 28 pg/ml, consistent with previously reported data [26, 27, 30]. Compared to the control group, serum sP2X7R concentrations in pregnant women were significantly higher in the first (141.5 ± 68 pg/ml; *p* = 0.0019), second (172.3 ± 81 pg/ml; *p* < 0.0001) and third trimester (190.7 ± 89 pg/ml; *p* < 0.0001) of pregnancy (Fig. 1a), confirming the inflammatory state typical of gestation. Comparison of sP2X7R concentrations between the three trimesters using Tukey’s test showed a significant increase between the first and second trimester (*p* = 0.0002) and between the first and the third trimester (*p* < 0.0001), while the difference between the second and the third trimester was not significant (Fig. 1a).

Recent findings have generated interest in exploring the use of certain haematological indices such as the neutrophil-to-lymphocyte ratio (NLR), calculated as the ratio of the absolute number of neutrophils to the absolute number of lymphocytes in peripheral blood, as a marker of systemic inflammation. Based on the observation that, during healthy pregnancy, the number of neutrophils in peripheral blood increases while the number of lymphocytes decreases [34, 35] several studies have analysed the NLR as a potential predictive marker of pregnancy complications [36–41]. Although reference intervals are not consistent across studies, most suggest an NLR reference interval between 1.5 and 2.2 in healthy non-pregnant women, and mean NLR values of 2.6 to 4.0 during normal pregnancy [35, 42]. The average NLR values in our cohorts are consistent with previous studies. We found mean NLR values of 1.55 ± 0.7 in the control group, increasing to 2.81 ± 1.2 in the first, 3.48 ± 1.2 in the second, and 3.54 ± 1.0 in the third trimester of pregnancy (Fig. 1b). The differences between control and pregnant women were highly significant (*p* < 0.0001) in all three trimesters; the differences between the first and the second trimester, and between the first and the third trimester, were also significant (*p* < 0.0001) (Fig. 1b).

Therefore, as the increase of sP2X7R parallels that of NLR during the three trimesters, sP2X7R can be considered as a marker of the inflammatory state typical of pregnancy.

### sP2X7R concentrations increase in multigravida and multiparous women

To further investigate the role of sP2X7R in different pregnancy conditions, we divided our cohort of pregnant women into primigravida (first pregnancy) and multigravida (second or subsequent pregnancy) groups.

**Fig. 2:**
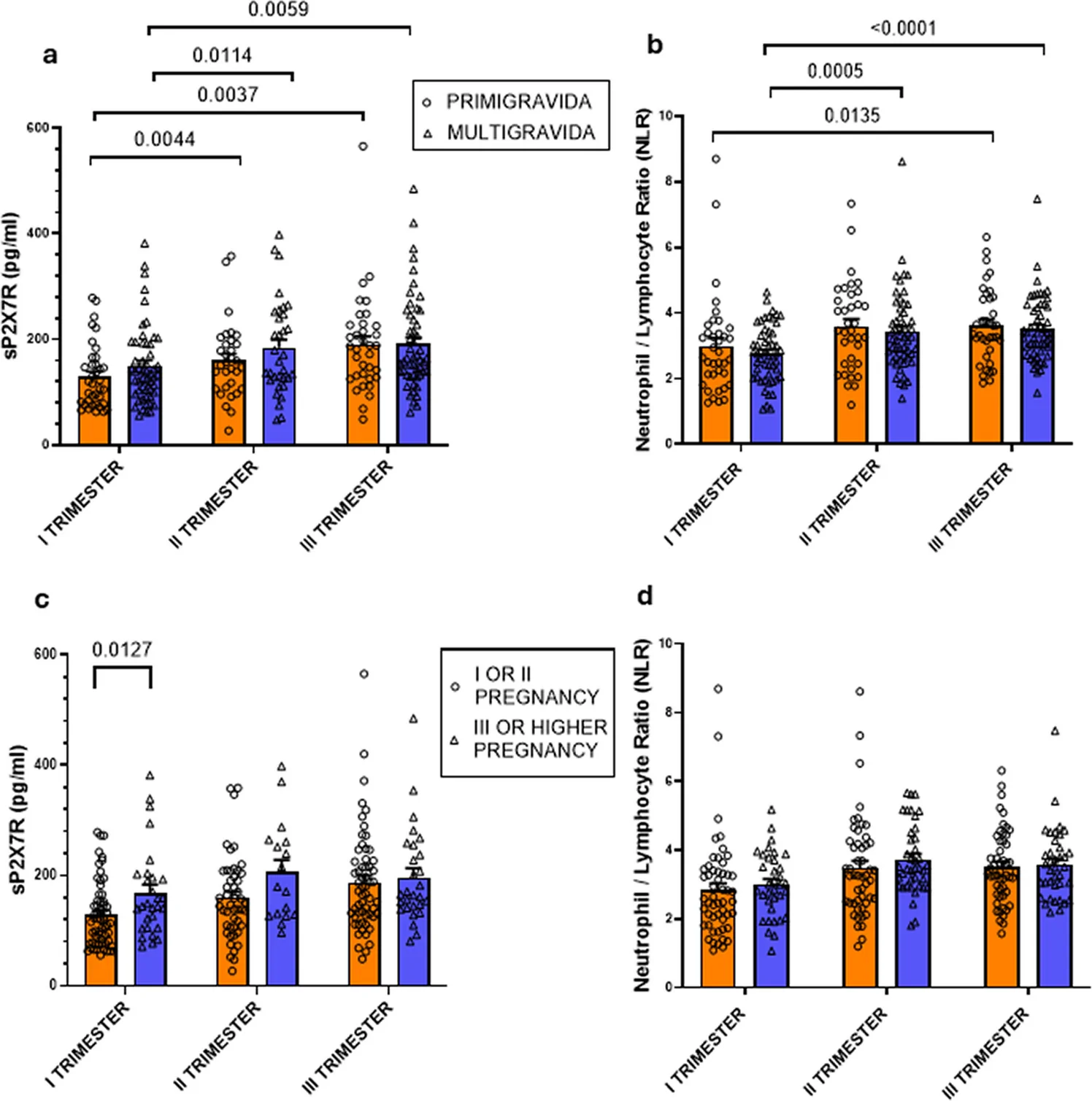
Serum sP2X7R concentrations and neutrophil-to-lymphocyte ratios (NLR) according to gravidity. Pregnant women were submitted to blood withdrawal at each trimester and serum sP2X7R and NLR were determined as described under methods. **a**, **c** Serum sP2X7R concentrations and **b**, **d** NLR values were measured in **a**, **b** primigravida (first pregnancy) and multigravida (second or subsequent pregnancy) women; **c**, **d** women in their first or second pregnancy and women in their third or subsequent pregnancy. sP2X7R ELISA measurements were performed in technical duplicates. Data are expressed as mean ± SEM. Statistical analysis was performed as described under Methods. Differences were considered statistically significant when *p* < 0.05

As shown in Fig. 1, serum sP2X7R increased during pregnancy in both primigravida (129.3 ± 60 pg/ml, 160.2 ± 70 pg/ml, 190.1 ± 91 pg/ml) and multigravida (150.1 ± 73 pg/ml, 184.0 ± 89 pg/ml, 191.2 ± 89 pg/ml) women (Fig. 2a). Amounts in the second and third trimesters were significantly higher than those in the first trimester in both primigravida (*p* = 0.0044 and *p* = 0.0037, respectively) and multigravida (*p* = 0.0114 and *p* = 0.0059, respectively) (Fig. 2a). NLR evaluation showed a significant increase across the three trimesters only in multigravida (II vs I trimester: 3.43 ± 1.2 vs 2.76 ± 0.9, *p* = 0.0005; III vs I trimester: 3.51 ± 0.9 vs 2.76 ± 0.9, *p* < 0.0001), while in the primigravida group, the significant increase was only between the first and the third trimester (2.98 ± 1.5 vs 3.61 ± 1.1, *p* = 0.0135) (Fig. 2b).

Although the differences were not significant, in each trimester the sP2X7R concentrations in multigravida women were higher than those in primigravida women, suggesting an involvement of sP2X7R in repeated pregnancies. To further investigate the significance of this phenomenon, we divided our cohort into two groups: one comprising women in their first or second pregnancy, and the other comprising women in their third or subsequent pregnancy. We found that in the first trimester sP2X7R concentrations were significantly higher in the second group than in the first group (168.3 ± 80 pg/ml vs 128.8 ± 58 pg/ml, *p* = 0.0127). Although the difference was not statistically significant, also during the second trimester sP2X7R concentrations remained higher in the second group compared with the first group (206.3 ± 90 pg/ml vs 160.2 ± 74 pg/ml, *p* = 0.073) (Fig. 2c). This highlights the greater sensitivity of the sP2X7R test, compared to the NLR, which showed no significant differences between the two groups (Fig. 2d), in detecting the inflammatory condition likely exacerbated by the total number of pregnancies. These observations suggest that sP2X7R provides additional information beyond that offered by NLR regarding the inflammatory response during pregnancy, particularly between primigravida and multigravida women.

**Fig. 3:**
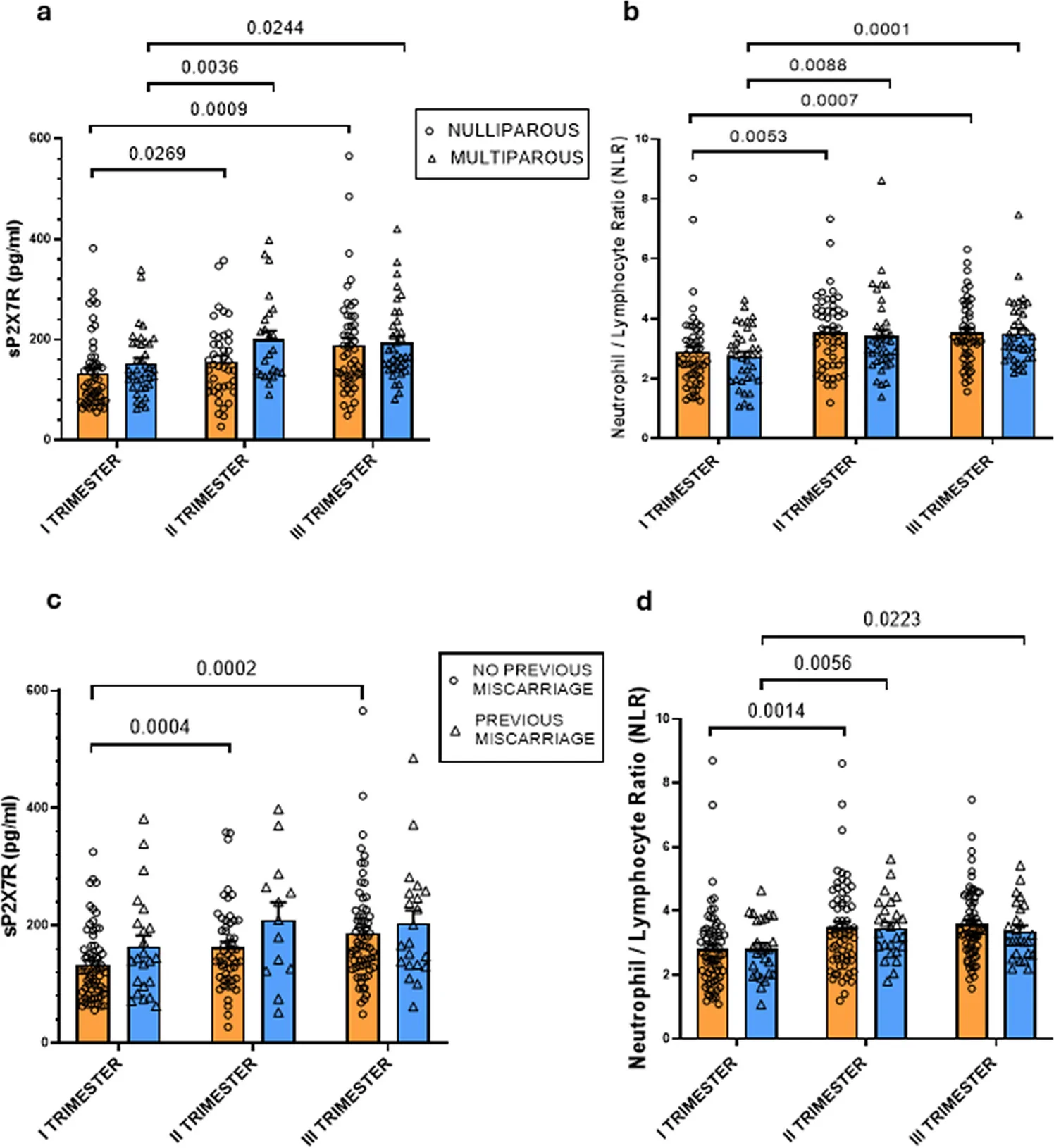
Serum sP2X7R concentrations and neutrophil-to-lymphocyte ratios (NLR) according to parity or previous miscarriages. Pregnant women were submitted to blood withdrawal at each trimester and serum sP2X7R and NLR were determined as described under methods. **a**, **c** Serum sP2X7R concentrations and **b**, **d** NLR values were determined in: **a**, **b** nulliparous and multiparous women, and **c**, **d** women with or without a previous history of miscarriage. Data are expressed as mean ± SEM. Statistical analysis was performed as described under Methods. Differences were considered statistically significant when *p* < 0.05

We further divided our cohort according to parity into nulliparous (no delivery yet) and multiparous (one or more previous deliveries) women. As expected, sP2X7R concentrations significantly increased across pregnancy in both nulliparous (II vs I trimester: *p* = 0.0269; III vs I trimester: *p* = 0.0009), and multiparous women (II vs I trimester: *p* = 0.0036; III vs I trimester: *p* = 0.0244) (Fig. 3a). Furthermore, we observed higher sP2X7R values—very close to statistical significance—in multiparous women compared to nulliparous women in the first (153.2 ± 63 pg/ml vs 133.4 ± 71 pg/ml, *p* = 0.053) and in the second trimester (200.0 ± 85 pg/ml vs 156.1 ± 74 pg/ml, *p* = 0.051) (Fig. 3a). Despite the lack of statistical significance, the higher concentrations observed in the multiparous group during the first and second trimesters might suggest an accentuated inflammatory state in multiparous women during the early stages of pregnancy. The NLR values increased over the three trimesters in both nulliparous (II vs I trimester: 3.53 ± 1.2 vs 2.88 ± 1.3, *p* = 0.0053; III vs I trimester: 3.53 ± 1.0 vs 2.88 ± 1.3, *p* = 0.0007), and multiparous women (II vs I trimester: 3.42 ± 1.3 vs 2.74 ± 0.9, *p* = 0.0088; III vs I trimester: 3.52 ± 1.0 vs 2.74 ± 0.9, *p* = 0.0001) (Fig. 3b). Unlike sP2X7R, regarding NLR, no relevant differences were found between nulliparous and multiparous women in any of the trimesters.

Given that spontaneous abortion has been associated with increased circulating cytokine levels [43–45]**,** we investigated whether spontaneous abortion also affects serum sP2X7R concentrations in pregnant women. As shown in Fig. 3c, in pregnant women without any previous miscarriage, sP2X7R concentrations increased significantly throughout the three trimesters (II vs I trimester *p* = 0.0004; III vs I trimester *p* = 0.0002), suggesting that in these women with no prior history of spontaneous abortion, the increase in sP2X7R is gradual and consistent during gestation. In contrast, in pregnant women who had experienced previous miscarriages the increases in sP2X7R concentrations across the three trimesters were not significant. Interestingly, although not significantly, sP2X7R amounts were higher in women who had experienced miscarriages than in those who had not at the first (164.6 ± 86 pg/ml vs 133.6 ± 59 pg/ml), second (209.7 ± 106 pg/ml vs 162.9 ± 71 pg/ml), and third trimester (203.8 ± 98 pg/ml vs 186.6 ± 87 pg/ml). It appears evident that pregnant women with a history of miscarriage exhibited higher sP2X7R concentrations already at the start of the new pregnancy, compared to the study population (164.6 ± 86 pg/ml vs 141.5 ± 68 pg/ml), although these concentrations showed a tendency to increase over the course of gestation.

No difference in NLR was found between the two groups in each trimester: 2.80 ± 1.3 vs 2.81 ± 0.9 in the first, 3.49 ± 1.4 vs 3.46 ± 0.9 in the second, and 4.21 ± 5.4 vs 3.37 ± 0.9 in the third trimester. However, unlike sP2X7R, NLR increased significantly throughout the three trimesters in women with previous miscarriage (II vs I trimester *p* = 0.0056; III vs I trimester *p* = 0.0223) and, like sP2X7R, between the first and the second trimester in women without miscarriage (*p* = 0.0014) (Fig. 3d).

### sP2X7R concentrations in pregnancy-associated pathologic conditions

Several studies have shown that obesity in pregnancy is linked to an increased risk of pregnancy complications, such as gestational hypertension, gestational diabetes and pre-eclampsia, with inflammatory mechanisms involved [46–48]. Obesity is associated with chronic systemic inflammation that affects female fertility by compromising oocyte quality, early embryonic development and endometrial receptivity [49]. Several studies report alterations in peripheral blood concentrations of various cytokines, such as IL-4, IL-10, IL-12, IL-13, IFN-γ, and TNF-α, as well as modifications of the follicular microenvironment in overweight and obese women [50, 51].

**Fig. 4:**
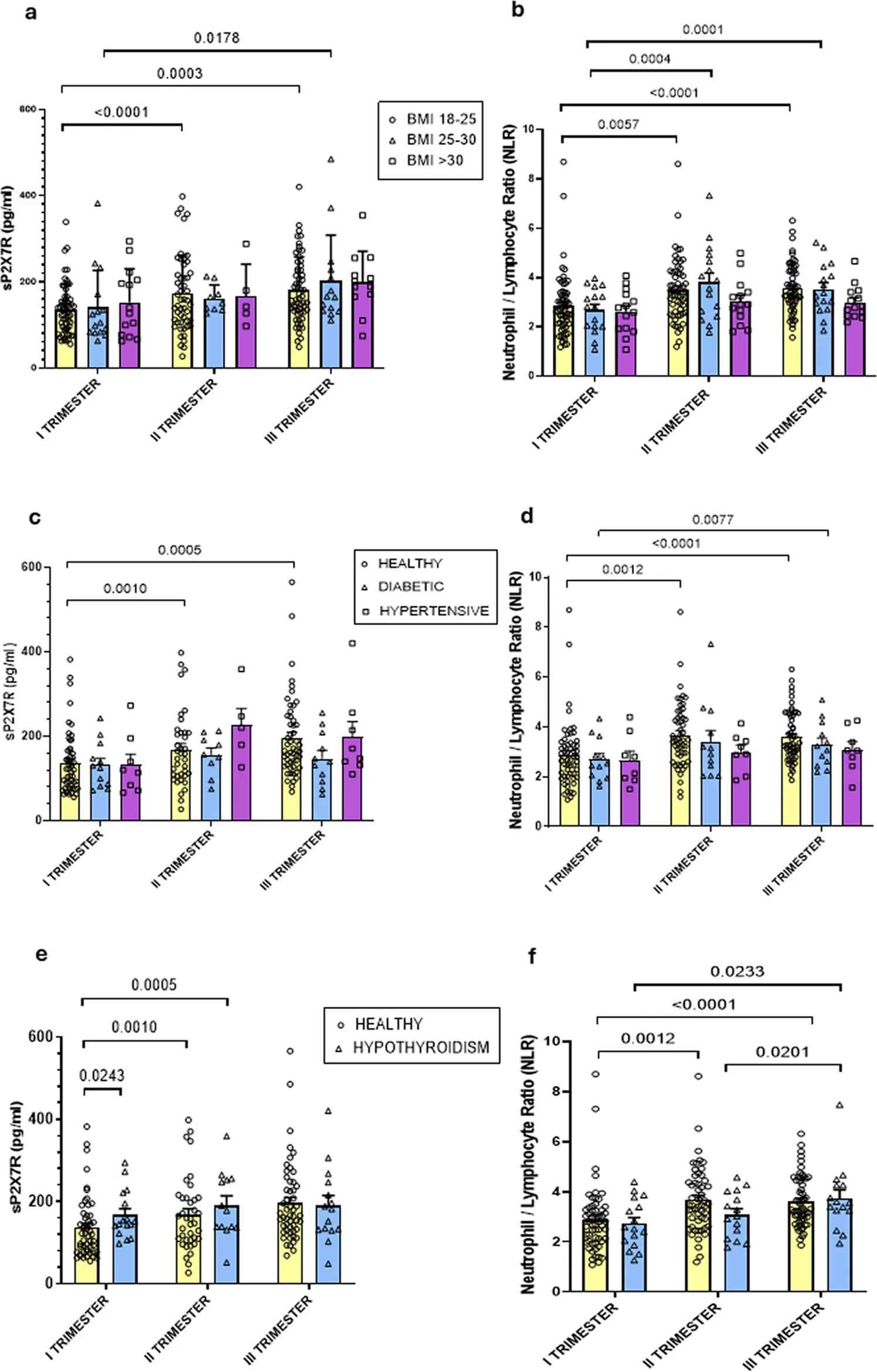
Serum sP2X7R concentrations and neutrophil-to-lymphocyte ratios (NLR) according to BMI and pregnancy-associated comorbidities. Pregnant women were submitted to blood withdrawal at each trimester and serum sP2X7R and NLR were determined as described under methods. **a**, **c**, **e** Serum sP2X7R concentrations and **b**, **d**, **f** NLR values were measured in **a**, **b** pregnant women stratified according to their Body Mass Index (BMI): 18–25, 25–30, and > 30 kg/m^2^; **c**, **d** healthy, diabetic and hypertensive pregnant women; **e**, **f** pregnant women either healthy or diagnosed with hypothyroidism. sP2X7R ELISA measurements were performed in technical duplicates. Data are expressed as mean ± SEM. Statistical analysis was performed as described under Methods. Differences were considered statistically significant when *p* < 0.05

In our study, we found that sP2X7R concentrations significantly increased throughout the three trimesters of pregnancy both in women with normal Body Mass Index (BMI = 18–25) (II vs I trimester: 172.7 ± 88 pg/ml vs 135.4 ± 58 pg/ml, *p* < 0.0001; III vs I trimester: 181.5 ± 76 pg/ml vs 135.4 ± 58 pg/ml, *p* = 0.0003), and in overweight women (BMI = 25–30) (III vs I trimester: 202.7 ± 105 pg/ml vs 140.3 ± 86 pg/ml, *p* = 0.0178). In obese women (BMI > 30), the increase in sP2X7R throughout the three trimesters (152.3 ± 78 pg/ml, 166.8 ± 74 pg/ml, 199.1 ± 71 pg/ml) was not significant (Fig. 4a). Moreover, no significant difference was found between the different groups in each trimester (Fig. 4a).

In agreement with sP2X7R, NLR increased over the three trimesters in both normal weight (II vs I trimester: 3.50 ± 1.3 vs 2.86 ± 1.3, *p* = 0.0057; III vs I trimester: 3.59 ± 1.0 vs 2.86 ± 1.3, *p* < 0.0001) and overweight women (II vs I trimester: 3.80 ± 1.5 vs 2.68 ± 0.9, *p* = 0.0004; III vs I trimester: 3.53 ± 1.0 vs 2.68 ± 0.9, *p* = 0.0001) (Fig. 4b). Interestingly, in obese women, as with sP2X7R, NLR also increased slightly over the three trimesters (2.62 ± 0.9, 3.04 ± 0.9, 2.99 ± 0.7) without any significant difference.

Gestational diabetes and hypertension are additional inflammatory conditions associated with pregnancy complications [52, 53]. Therefore, we compared women with gestational diabetes or gestational hypertension with healthy pregnant women. Rather surprisingly, diabetic women did not show any relevant increase in sP2X7R across the three trimesters (132.3 ± 55 pg/ml, 156.6 ± 48 pg/ml, and 147.5 ± 63 pg/ml, respectively) (Fig. 4c).

In contrast, women with hypertension showed increasing sP2X7R values across the three trimesters (133.0 ± 70 pg/ml, 226.6 ± 87 pg/ml and 199.0 ± 101 pg/ml, respectively), which were however very similar to those of healthy pregnant women (136.9 ± 74 pg/ml, 168.4 ± 89 pg/ml, and 196.3 ± 96 pg/ml, respectively) (Fig. 4c). Only women with healthy pregnancies, free from both diabetes and hypertension, showed significant increases in sP2X7R concentrations (II vs I trimester *p* = 0.0010; III vs I trimester *p* = 0.0005) (Fig. 4c).

The NLR values increased over the three trimesters in healthy pregnant women (II vs I trimester: 3.67 ± 1.3 vs 2.87 ± 1.3, *p* = 0.0012; III vs I trimester: 3.61 ± 1.0 vs 2.87 ± 1.3, *p* < 0.0001) and in diabetic women (III vs I trimester: 3.28 ± 0.9 vs 2.68 ± 0.8, *p* = 0.0077) (Fig. 4d).

As numerous studies have reported an association between hypothyroidism and various pregnancy disorders such as preeclampsia, miscarriage and preterm birth [54, 55], we investigated sP2X7R amounts in pregnant women with hypothyroidism. As shown in Fig. 4e, in addition to the expected significant increase in sP2X7R in the second and third trimesters compared to the first trimester (*p* = 0.0010 and *p* = 0.0005, respectively), sP2X7R was also significantly higher in pregnant women with hypothyroidism than in healthy pregnant women (168.0 ± 58 pg/ml vs 136.9 ± 74 pg/ml, *p* = 0.0243) during the first trimester.

Regarding NLR, healthy women showed significant increases in the second and third trimesters compared to the first (*p* = 0.0012 and *p* < 0.0001), while women with hypothyroidism showed increased values between the first and third trimesters (*p* = 0.0233), and between the second and third trimesters (*p* = 0.0201). No significant difference was found between healthy and hypothyroid pregnant women in any trimester (Fig. 4f).

### Maternal sP2X7R concentrations increase with male fetus and cross the placenta

**Fig. 5:**
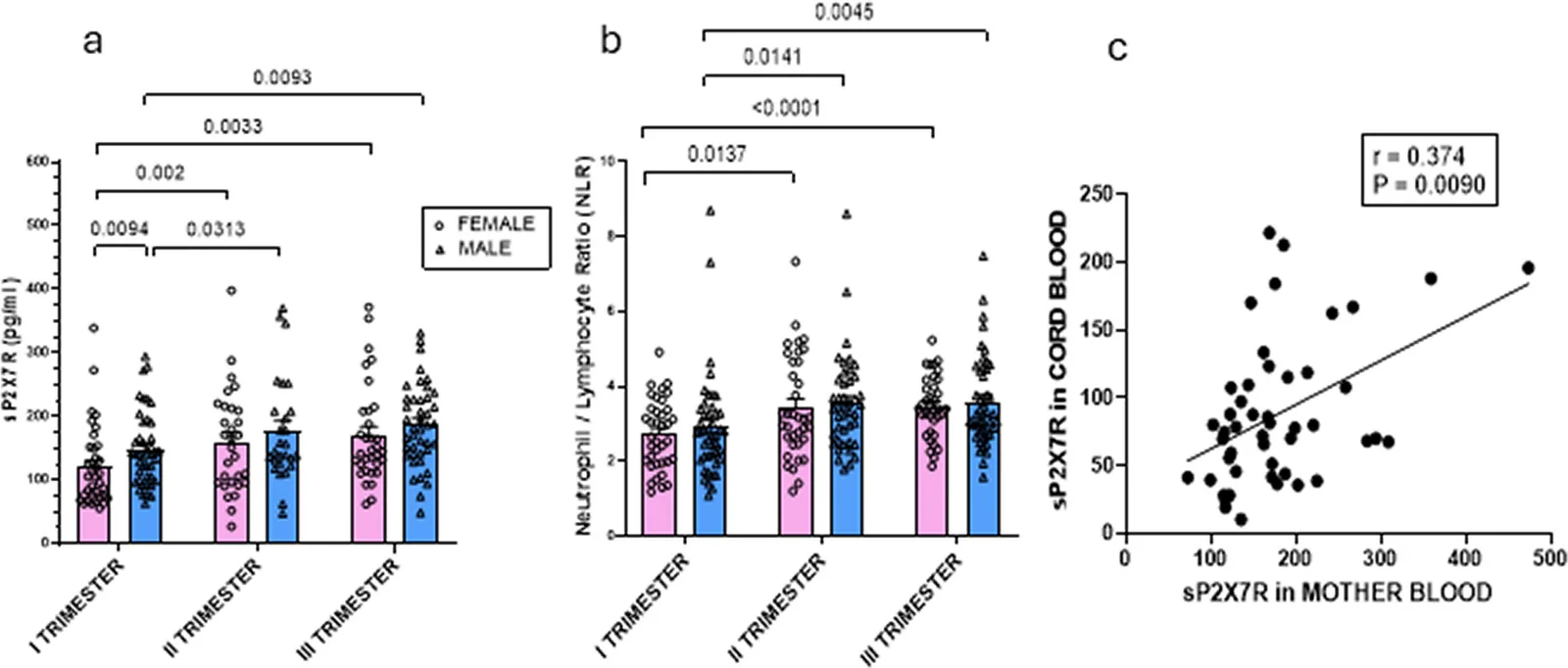
Serum sP2X7R concentrations and neutrophil-to-lymphocyte ratios (NLR) according to fetal sex and correlation between maternal and cord blood sP2X7R. **a**, **b** Maternal serum sP2X7R concentrations and NLR values in pregnant women carrying either female or male fetus. **c** Correlation between maternal and cord blood sP2X7R concentrations assessed using Spearman’s correlation test (*r* = 0.374, *p* = 0.0090). sP2X7R ELISA measurements were performed in technical duplicates. Data are expressed as mean ± SEM. Statistical significance between groups was assessed using the nonparametric Mann–Whitney U test. Differences were considered statistically significant when *p* < 0.05

Previous studies have reported that male fetuses are associated with a strong pro-inflammatory state and a low maternal plasma anti-inflammatory cytokine profile [56, 57]. In agreement with these observations, we found higher maternal sP2X7R concentrations in pregnancies with male fetuses than in those with female fetus during the first trimester (148.5 ± 58 pg/ml vs 121.0 ± 63 pg/ml, *p* = 0.0094) (Fig. 5a), the most critical period of pregnancy. Conversely, NLR does not appear to be as sensitive as sP2X7R, as it did not show any significant differences between male and female fetuses (2.90 ± 1.3 vs 2.72 ± 0.9) (Fig. 5b).

In a healthy pregnancy, there is continuous bidirectional communication between the maternal and fetal compartments mediated by the placental circulation. This allows the exchange of nutrients and metabolic waste products between mother and fetus, as well as cells and signalling molecules. Moreover, the placenta facilitates the transfer of microvesicles, exosomes, and cell-free (cf) components, including proteins, RNA (cfRNA), and DNA (cfDNA), thereby supporting dynamic maternal–fetal interaction [58]. Although the umbilical cord has received less attention than other reproductive tissues, it is a valuable source of endogenous biomarkers associated with pregnancy outcomes. Studies have shown that cord blood contains molecular signatures indicative of various fetal and neonatal conditions, including immune activation, sepsis, gestational diabetes–associated macrosomia, and fetal-specific immune responses [59–61]. In our study, blood samples collected from umbilical cords at birth were used to measure sP2X7R concentrations (mean 87.9 ± 54 pg/ml). A significant positive correlation was found between sP2X7R concentrations in maternal and cord blood (*r* = 0.374, *p* = 0.0090) (Fig. 5c), suggesting that sP2X7R likely crosses the placenta and reaches the fetal circulation.

## Discussion

Pregnancy is a complex natural phenomenon in which the maternal body’s adaptations are determined by the controlled and coordinated production of various hormones, cytokines and other factors within the maternal-fetal interface [62–64]. In general, immune regulation in a pregnant mother is governed by innate immune responses and a tight balance between adaptive Th1 responses, characterized by pro-inflammatory cytokines such as IL-1, IFN-γ and TNF-α, and Th2 responses, with typical anti-inflammatory cytokines like IL-10, IL-5 and IL-4 [65, 66].

During pregnancy, three distinct immunological stages have been described based on the body’s inflammatory response: an initial pro-inflammatory stage, important for implantation and placentation; a Th2 anti-inflammatory response, necessary for fetal growth during the second trimester; and finally, a Th1-mediated stage, crucial for initiating labour and delivery [67]. Over the years, several studies based on circulating cytokines and immune cell profiles have supported the hypothesis that normal pregnancy is predominantly a Th2-phenomenon, while excessive pro-inflammatory responses have been associated with various adverse perinatal health outcomes, including preterm delivery, altered fetal development, and pregnancy complications such as gestational diabetes, gestational hypertension and preeclampsia [14, 68–70]. Cytokine levels appear to predict pregnancy conditions, and there is growing interest in describing typical variations in immune parameters throughout pregnancy and identifying relevant deviations. Nonetheless, few studies have at present examined longitudinal changes in circulating cytokines as pregnancy progresses [71, 72].

Extracellular ATP (eATP) is the earliest and most ubiquitous damage-associated molecular pattern (DAMP) released at inflammatory sites. Among the various eATP receptors, P2X7R is particularly relevant for its ability to initiate the inflammatory cascade and promote disease progression by regulating T-cell and adaptive immune responses, strongly stimulating the release of inflammatory cytokines [73, 74]. Several studies have shown that activation of the P2X7R/NLRP3 inflammasome pathway is involved in the pathogenesis of preeclampsia and other pregnancy syndromes associated with placental inflammation [24, 25].

P2X7R regulates ATP-induced uterine contraction, and its expression is associated with successful labour and preterm delivery [75, 76]. Studies have demonstrated that pharmacological blockade of maternal P2X7R prevents preterm birth and ameliorates perinatal brain injury following exposure to intrauterine inflammation [24, 77]. Activation of the maternal immune system during pregnancy acts as an early priming event that sensitizes the fetal brain, resulting in transcriptional and behavioural changes in offspring through cytokine signalling mechanisms [78, 79].

Recently, a soluble form of the P2X7R (sP2X7R) has been shown to be present in blood at elevated amounts in several inflammatory diseases and has been tentatively added to the range of inflammatory mediators [26, 80]. As the maternal serum sP2X7R may reflect various immunological processes in the body, we hypothesized that circulating sP2X7R concentrations might represent a reliable and easily measurable indicator of the extent of P2X7R activation during pregnancy, especially in the presence of inflammatory complications.

First, the controls used in this study, represented by sera from 30 healthy non-pregnant women of low–intermediate age (around 30–35 years), showed sP2X7R concentrations similar, though slightly higher, than those reported in previous studies [26, 27, 30]. In our experience we have observed a certain degree of variability in sP2X7R concentrations, depending on the subject’s age and sex, the use of plasma or serum, and sample storage conditions. Evidently, the conditions regarding sex, age, and sample type used in this study selected samples with higher sP2X7R contents. Nevertheless, the comparison with pregnant women, who presumably present a heightened inflammatory state, remains significant. Interestingly, sP2X7R concentrations were not only significantly higher in pregnant women compared to non-pregnant controls but also increased across the three trimesters of gestation.

The increment in sP2X7R was observed both in women in their first pregnancy (primigravidae) and in those in their second or subsequent pregnancy (multigravidae). Although not statistically significant, multigravidae showed sP2X7R concentrations higher than those of primigravidae. We therefore separately analysed women in their first or second pregnancy and those in their third or subsequent pregnancy, finding that sP2X7R concentrations were higher in the second group than in the first during the first trimester. These results are consistent with previous observations of stronger immune activation in multiparous women in their first trimester of pregnancy [81]. One possible explanation is that multigravid women may have been sensitized to fetal antigens during a previous pregnancy and developed an immune response against trophoblast antigens in a subsequent pregnancy, triggering an inflammatory reaction during the early stages of gestation.

Both nulliparous and multiparous women showed significantly increased amounts of sP2X7R across the three trimesters. Furthermore, in the first and second trimesters we observed higher sP2X7R concentrations, very close to statistical significance, in multiparous compared to nulliparous women, suggesting an accentuated inflammatory state in multiparous women during the early stages of pregnancy.

Conversely, when we stratified our cohort based on the presence or absence of prior miscarriages, unlike women with no history of miscarriage, those with a prior pregnancy loss showed no significant increase in sP2X7R concentrations across the three trimesters. It is interesting to note that these women, compared with those with no history of miscarriage, already exhibited higher, albeit not statistically significant, amounts of circulating sP2X7R during the early stages of pregnancy. This latter observation suggests that sP2X7R may exhibit a profile like that of pro-inflammatory cytokines, whose concentrations were elevated during the early stages of pregnancy in women who subsequently experienced a miscarriage or with  a history of pregnancy loss [82–84]. Although the clinical literature linking P2X7R to spontaneous abortion is limited compared with studies on cytokines, available evidence suggests that excessive P2X7R-mediated signaling could contribute to early pregnancy failure [85].

Several studies in the literature have shown that mothers with pre-pregnancy obesity exhibit elevated concentrations of pro-inflammatory cytokines and mediators, such as IL-8, IL-6, CRP and TNF-α [86–89]. Obesity and overweight can adversely affect female fertility by compromising oocyte quality and early embryonic development [49]. Rather surprisingly, our data do not show significantly higher concentrations of sP2X7R in overweight or obese women compared with normal-weight women. However, due to various factors—such as biological variability, the type of sample (serum versus plasma), or the mothers' fasting status—variations in cytokine levels can also appear inconsistent [24].

In women with gestational diabetes, sP2X7R concentrations did not differ statistically significantly from those of healthy pregnant women, and the trend towards an increase in sP2X7R during pregnancy was very slight. In contrast, hypertensive women—albeit a small subgroup of our cohort (8 out of 90)—showed rising sP2X7R values across the three trimesters, values very similar to those observed in healthy pregnant women. A larger sample of subjects could, however, enhance the reliability of these findings. The question remains whether altered metabolic and inflammatory conditions might indirectly adversely affect fetal growth by impairing various placental functions, despite the limited utility of measuring sP2X7R in this context. This result could be attributed to a potentially greater sensitivity of sP2X7R to acute inflammation, such as that associated with pregnancy itself, compared to the chronic inflammatory conditions linked to obesity, diabetes, or hypertension.

Another common clinical issue during pregnancy is hypothyroidism [90]. High maternal TSH concentrations have been associated with dysregulated cytokine concentrations, pregnancy complications, and adverse neurocognitive outcomes in offspring [91–93]. Our results show that, in the first trimester, sP2X7R concentrations are higher in pregnant women with hypothyroidism than in healthy pregnant women. To the best of our knowledge, our data provides the first evidence that sP2X7R may serve as an inflammatory biomarker in gestational hypothyroidism.

We also found that the presence of a male fetus is associated with higher concentrations of sP2X7R in the first trimester, a finding consistent with previous results indicating that male fetuses appear to increase maternal cytokine levels [57].

At present, the mechanisms underlying sP2X7R generation have not yet been identified and it has not yet been determined whether the circulating forms of P2X7R are exosome-associated, the full-length protein, and/or fragments generated by proteolytic cleavage. Given the wide distribution of P2X7R across different tissues in the body, the soluble form of the receptor can, in principle, originate from various cell types. One possible source can be circulating white blood cells, likely in an activated state, as well as inflamed tissues or tumours. It is highly likely that circulating sP2X7R concentrations depend strictly on the specific cell population involved in its release, as well as on the activation state of these cells. In our original study [26], we demonstrated that sP2X7R was released from unstimulated human blood monocytes, and that this release more than doubled following stimulation with BzATP.

One possible mechanism for P2X7R release from cells or tissues could involve the activity of proteolytic enzymes, such as matrix metalloproteinases (MMPs). This hypothesis is supported by the finding that sustained P2X7R activation triggers the release of MMP-2, which, by causing proteolytic receptor cleavage, terminates ion channel and macropore responses, thereby initiating a protective mechanism that allows cells with high P2X7R expression to escape ATP-induced cytotoxicity [94]. Various P2XRs, including P2X7R, and MMPs, including MMP-2, are expressed by the placenta and other maternal tissues [95, 96]. Furthermore, their presence in tissues and blood is increased in pregnancy-related disorders, such as preeclampsia, trophoblastic diseases and recurrent pregnancy loss [96–98]. Given that here we found that sP2X7R concentrations are more closely correlated with pregnancy itself than with the mother's general condition (BMI, diabetes, hypertension), it is highly likely that sP2X7R originates from maternal tissues and that its variations could be exploited to identify pregnancy-related disorders.

We had previously demonstrated that at least a fraction of plasma sP2X7R was associated with MVs/MPs as plasma depleted of MVs/MPs via ultracentrifugation (100000 × g for 90 min at 4 °C) showed reduced sP2X7R concentrations compared to unprocessed whole plasma. Although the MVs/MPs were not characterized in terms of number and size, the presence of the receptor, demonstrated by Western blot analysis [26], further supports the role of these subcellular fractions in the dissemination of the receptor itself. It is noteworthy that exosomes have been detected in the placenta at high concentrations in the presence of pregnancy complications, such as preeclampsia and preterm birth, and are likely to provide inflammatory signaling molecules [69, 99, 100]. Exosomes have also been identified in amniotic fluid, where enrichment in inflammatory cytokines has been observed in the presence of intra-amniotic inflammatory conditions [69, 100]. Furthermore, fetal exosomes have been shown to be released into maternal tissues, likely activating inflammatory signaling pathways [101]. In our study, we detected the circulating form of P2X7R in umbilical cord blood samples and found a positive correlation between sP2X7R concentrations in maternal and cord blood. These data suggest that maternal inflammatory conditions could lead to increased amounts of inflammatory mediators in the developing fetus.

A growing body of evidence demonstrates that an elevated neutrophil-to-lymphocyte ratio (NLR) is associated with various pregnancy complications, including preeclampsia, gestational diabetes, gestational hypertension, and spontaneous preterm birth, confirming its potential role as an easily accessible inflammatory biomarker in obstetric care. Our findings are consistent with mean NLR values reported in the literature, which indicate that values below 2.5 are typically observed in healthy non-pregnant women, values between 2.5 and 4.5 are common in physiological pregnancies, and values exceeding 4.5–5 are associated with increased inflammatory activation and complications such as gestational diabetes, gestational hypertension, preeclampsia, and spontaneous preterm birth [36–41, 102, 103]**.** In our cohort, sP2X7R showed a pattern of variation comparable to that of NLR throughout pregnancy, while providing additional information on pregnancy-related inflammatory responses, particularly in multiparous women, those with hypothyroidism, and those carrying a male fetus.

In conclusion, despite the relatively small sample size, this is the first study demonstrating the potential of circulating sP2X7R as a novel biomarker of the inflammatory state that characterizes pregnancy and its associated pathological conditions.

## Data Availability

All data supporting the findings of this study are available within the paper.
